# A Multifunctional Nanodelivery System Modified by Fusion Peptides Acts as Teriparatide Carrier for Noise‐Induced Hearing Loss Therapy

**DOI:** 10.1002/advs.202408798

**Published:** 2025-02-07

**Authors:** Jiawen Li, Zhuowen Hao, Fangzi Ke, Zhihui Liu, Weilong Wang, Sihui Wen, Fan Wu, Bowen Xu, Miao He, Shengyu Zou, Xiong Chen, Jingfeng Li, Zuhong He

**Affiliations:** ^1^ Department of Otorhinolaryngology‐Head and Neck Surgery Zhongnan Hospital of Wuhan University Wuhan 430071 China; ^2^ Department of Spine Surgery and Musculoskeletal Tumor Zhongnan Hospital of Wuhan University Wuhan 430071 China; ^3^ Department of Pathology and Laboratory Medicine the Medical University of South Carolina Charleston 29425 USA

**Keywords:** fusion peptide, hearing loss, penetration, targeted drug delivery, teriparatide

## Abstract

Noise‐induced hearing loss (NIHL) is among the most poorly treated diseases due to irreversible damage to hair cells. Reactive oxygen species contribute to NIHL pathogenesis by injuring the inner ear hair cells. Teriparatide (PTH1‐34) exerts antioxidant properties in the context of osteoporosis. Thus, in the current study, a multifunctional thermosensitive nanodelivery system is developed, based on the unique anatomical structure of the inner ear, to load and sustain the release of PTH1‐34 to alleviate hair cell damage. LR27—a fusion peptide—is assembled from a targeting peptide (A665) and cell‐penetrating peptide (Arg8) to target hair cells and increase the round window membrane permeability. In vitro, the antioxidant and anti‐apoptotic properties of the nanodelivery system are assessed after treating HEI‐OC1 cells and cochlear explants with hydrogen peroxide to simulate the oxidative environment. In vivo, injection of the nanodelivery system into the auditory bulb protects the hearing and hair cells of an NIHL mouse model. These results demonstrate the synergistic effects of multiple peptides in treating NIHL and their potential clinical applications.

## Graphical abstract

The fusion peptide LR27‐modified thermosensitive nanodelivery system exhibits both hair cell targeting and inner ear penetrating properties. This system sustainably and effectively delivers PTH1‐34 to the inner ear of a hearing loss mouse model via the synergistic effects of multiple peptides, achieving satisfactory hearing protection through antioxidant and anti‐apoptotic properties.

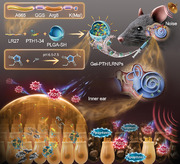

## 1 Introduction

Noise‐induced hearing loss (NIHL) is a sensory deafness due to noise exposure that has considerable negative impacts on quality of life and a globally increasing incidence.^[^ ^1^ ^]^ Although hearing aids and cochlear implants are the most useful interventions for rebuilding hearing, many patients cannot take advantage of these methods due to the difficulty of speech perception in noise.^[^ ^2^ ^]^ Moreover, no drug has been approved by the Food and Drug Administration (FDA) for treating NIHL, leaving steroids as the only clinical option for managing NIHL, although their efficacy is unsatisfactory.^[^ ^3^ ^]^

NIHL pathogenesis is complex, involving environmental and genetic factors that impact numerous molecular pathways.^[^ ^4^ ^]^ Sensory hair cells lack regenerative abilities; thus, their loss typically causes irreversible hearing damage.^[^ ^5^ ^]^ Overproduction of reactive oxygen species (ROS) in the mitochondria in response to noise exposure disrupts redox homeostasis, causing mitochondrial dysfunction and hair cell apoptosis.^[^ ^6^ ^]^ Therefore, decreasing ROS production to maintain hair cell homeostasis is a potential NIHL therapeutic strategy.^[^ ^7^ ^]^ Accordingly, antioxidants,^[^ ^8^ ^]^ pharmaceutical compounds,^[^ ^9^ ^]^ and large biological proteins,^[^ ^10^ ^]^ have been developed to remove ROS in NIHL models. Meanwhile, small‐molecule active peptides have high biosafety profiles, excellent degradation, low cost, ease of synthesis, and multiple biological functions.^[^ ^11^ ^]^ However, few studies have evaluated the efficacy of small‐molecule active peptides as NIHL therapeutics.

Parathyroid hormone (PTH) is a peptide secreted by the parathyroid gland.^[^ ^12^ ^]^ Teriparatide, also known as PTH1‐34, is a small polypeptide comprising the first 34 amino acids at its N‐terminal end of the PTH polypeptide.^[^ ^13^ ^]^ Given the role of PTH in calcium and phosphorus metabolism, PTH1‐34 has been widely evaluated in orthopedics.^[^ ^14^ ^]^ Specifically, PTH1‐34 enhances osteoporotic osseointegration by promoting human umbilical vein endothelial cell proliferation by inhibiting ROS generation and reducing mitochondrial damage.^[^ ^15^ ^]^ Considering the antioxidant properties of PTH1‐34, its delivery to the inner ear to alleviate NIHL is feasible. However, the role of PTH1‐34 in hearing therapy has not yet been reported.

Due to the specific anatomical location of the cochlea in the inner ear and the presence of the blood–labyrinth barrier, drug absorption is limited.^[^ ^16^ ^]^ Thus, intratympanic administration is clinically used to achieve local drug delivery with minimal side effects and maximum drug concentration.^[^ ^17^ ^]^ The round window membrane (RWM) and oval window are the only two permeable barriers between the middle and inner ear. Since the oval window is covered by the stapes base plate, the RWM is the main route by which drugs enter the inner ear.

Poly (lactic‐co‐glycolic acid) (PLGA) nanoparticles can across RWM into the inner ear;^[^ ^18^ ^]^ however, their transport efficiency is limited. Recent research has sought to address this issue by modifying the nanoparticle surface. For example, peptide A665, targeting the hair cell Prestin protein, increases the delivery efficiency of nanoparticles,^[^ ^19^ ^]^ while the cell‐penetrating peptide Arg8 increases delivery efficiency by enhancing RWM permeability.^[^ ^20^ ^]^ These strategies have improved drug delivery efficiency but exhibit limited functionality. Therefore, multifunctional nanoparticles are needed to achieve drug delivery to the inner ear. Fusion peptides represent a potential strategy;^[^ ^21^ ^]^ however, they have not been applied in otology. Moreover, prolonging the contact time between nanoparticles with the RWM and reducing clearance from the eustachian tube considerably can also increase delivery efficiency. Thermosensitive hydrogels allow the nanoparticles to be injectable and can remain in the middle ear to better penetrate the RWM for NIHL therapy.^[^ ^22^ ^]^ In particular, the triblock copolymer F127 hydrogel has stable thermosensitive properties, good biocompatibility, and soft texture.^[^ ^23^ ^]^ Thus, F127 may be suitable for middle ear injection; however, it has not yet been investigated in this context.

**Figure 1:**
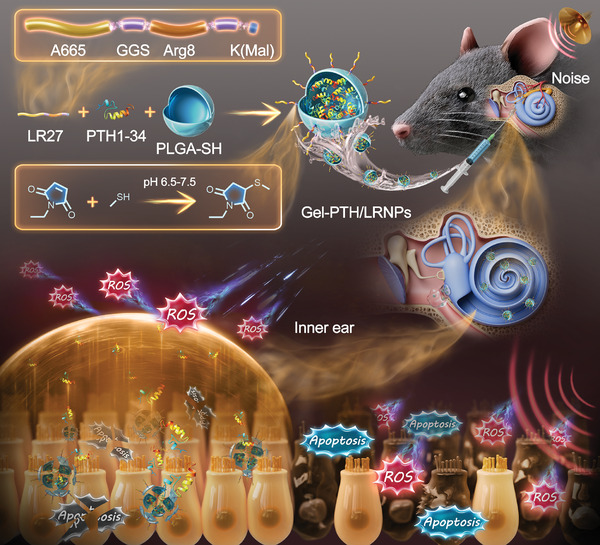
Schematic diagram of the multifunctional nanodelivery system modified by fusion peptide as a PTH1‐34 carrier for NIHL therapy. The fusion peptide LR27‐modified thermosensitive nanodelivery system exhibits hair cell targeting and inner ear penetrating properties. This system effectively delivers PTH1‐34 to the inner ear of a hearing loss mouse model via the synergistic effects of multiple peptides, achieving satisfactory hearing protection through antioxidant and anti‐apoptotic properties.

In the current study, a multifunctional nanodelivery system is designed for NIHL therapy. Sulfhydryl‐modified PLGA (PLGA‐SH) nanoparticles (PLGA‐SH nanoparticles, NPs) loaded with PTH1‐34 are prepared using a double emulsification method named PTH/NPs. The fusion peptide LR27, inspired by A665 and Arg8, is named based on its initial amino acid of functional sequences (L from A665 and R from Arg8) and the number of amino acids (27), combines targeting and penetration and is employed to modify PTH/NPs through an addition reaction between maleimide at the end of the fusion peptide and the sulfhydryl group on the PTH/NPs surface, creating PTH/LRNPs. Subsequently, the PTH/LRNPs are dispersed into the F127 thermosensitive hydrogel to create the nanodelivery system named Gel‐PTH/LRNPs. The antioxidant and anti‐apoptotic effects of PTH1‐34 are assessed in HEI‐OC1 cells and cochlear explants. Finally, the hearing‐protective effects of Gel‐PTH/LRNPs are evaluated in a mouse model of NIHL (**Figure** **1**). Thus, this study demonstrates the potential of small‐molecule active peptides for NIHL treatment and describes the design and assembly of a fusion peptide to expand the repertoire of inner ear treatment modalities.

## 2 Results and Discussion

### 2.1 Assembly and Characterization of Peptides and Nanoparticles

Nanoparticle delivery efficiency has been improved by adding amino acid sequences to the hair cell targeting‐peptide A665 (LSTHTTESRSMV) to facilitate its binding to different nanoparticles, such as PrTP1(LSTHTTESRSMVGGSCGGS[K(N_3_)])^[^ ^24^ ^]^ and LS19 (LSTHTTESRSMVGGSCGGS).^[^ ^25^ ^]^ Here, A665 was added with different amino acid sequences to obtain another hair cell‐targeting peptide LS16 (LSTHTTESRSMVGGS[K(Maleimide)]) and the fusion peptide LR27 (LSTHTTESRSMVGGSRRRRRRRRGGS[K(Maleimide)]), LS16 was named by combining its initial two amino acids of functional sequence (LS from A665) with the number of amino acids (16) and LR27 was named as the manner mentioned before. To achieve targeting and permeation effects, LR27 was assembled from A665 and the cell penetrating‐peptide Arg8 (RRRRRRRR) by the flexible linker (GGS). The lysine (K) at the end of LS16 and LR27 sequences were linked to maleimides (Table S1, Supporting Information). The successful synthesis of LS16 and LR27 was verified by high‐performance liquid chromatography (HPLC) and mass spectrometry (MS). HPLC analysis revealed that the purities of LS16 and LR27 were 95.16% and 96.42%, respectively (Figure S1, Tables S2 and S3, Supporting Information). MS analysis calculated the observed molecular weights of LS16 and LR27 to be 1828.95 and 3279.75 g mol^−1^, respectively, based on the m/z values of the molecular [M + nH] nH ions they produced. The theoretical molecular weight of LS16 and LR27 were 1828.96 and 3279.61 g mol^−1^, respectively (Figure S2, Supporting Information).

The maleimides at the ends of the LS16 and LR27 sequences formed stable thioether bonds with nanoparticles modified by sulfhydryl groups via an addition reaction at pH 6.5–7.5. When LR27 covalently binds nanoparticles modified by sulfhydryl groups, the hair cell‐targeting region of LR27 is exposed to the outermost part and functions normally. The positively charged cell‐penetrating peptide Arg8, located in the middle part, on its lateral side acts through nonspecific electrostatic aggregation with the negatively charged RWM.^[^ ^26^ ^]^ The hair cell targeting‐peptide coupled to the surface of nanoparticles, as catalyzed by N‐(3‐dimethylaminopropyl)‐N'‐ethylcarbodiimide (EDC) and N‐hydroxysuccinimide (NHS) in previous methods.^[^ ^27^ ^]^ However, PTH1‐34 could covalently bind the surface of nanoparticles by EDC and NHS as well, impacting the release and activity of PTH1‐34. To avoid this, the reaction between maleimide and sulfhydryl groups was selected to achieve peptide binding to the nanoparticles. Given that PTH1‐34 does not have maleimide, it does not couple to the nanoparticles during the reaction.

**Figure 2:**
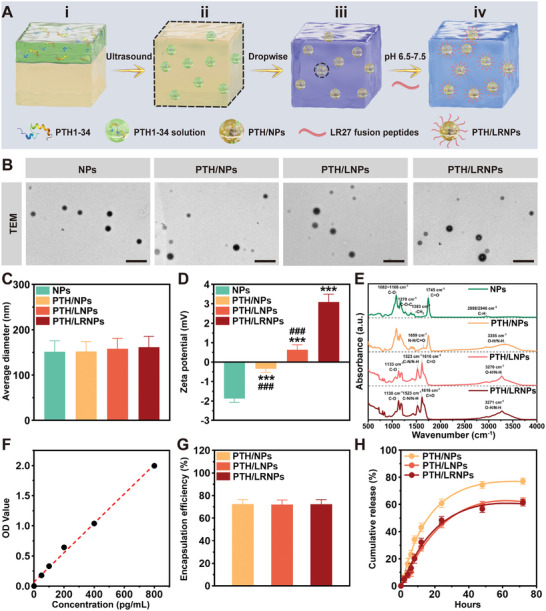
Preparation and characterization of different nanoparticle groups. A) Schematic diagram of PTH/LRNPs fabrication. (i) PTH1‐34 solution (green) and DCM with PLGA‐SH dissolved (yellow); (ii) The emulsion was obtained from (i) after ultrasound; (iii) added emulsion (ii) dropwise to the PVA solution (purple) to obtain PTH/NPs. The black dotted line indicates the dropwise addition process; (iv) Collected PTH/NPs and placed them in pH 7.4 PBS solution (blue) with LR27 dissolved. PTH/NPs and LR27 were covalently combined to obtain PTH/LRNPs. B) TEM images of four nanoparticle groups, scale bar: 500 nm. C) Average diameter of nanoparticles. D) Zeta potential of nanoparticles. E) FTIR analysis of nanoparticles. F) Standard curve of PTH1‐34. G) Encapsulation efficiency of PTH1‐34 by three nanoparticle groups. H) Release kinetics of PTH1‐34 from three nanoparticle groups. Data are expressed as mean ± standard deviation (SD; *n* = 3). ^***^ *p* < 0.001 compared with the NPs group; ^###^ *p* < 0.001 compared with the PTH/LRNPs group; DCM: dichloromethane, PLGA‐SH: sulfhydryl‐modified poly (lactic‐co‐glycolic acid), PVA: polyvinyl alcohol, PBS: phosphate buffered saline.

Nanoparticles were prepared using a double emulsification method (**Figure** **2**A). PLGA‐SH was selected as the nanoparticle. PTH1‐34, as an internal aqueous phase, was successfully loaded into PLGA‐SH via ultrasound. The nanoparticles were assigned to four groups: 1) NPs, ddH_2_O‐loaded PLGA‐SH nanoparticles; 2) PTH/NPs, PTH1‐34‐loaded PLGA‐SH nanoparticles; 3) PTH/LNPs, PTH1‐34‐loaded PLGA‐SH nanoparticles modified by LS16; 4) PTH/LRNPs, PTH1‐34‐loaded PLGA‐SH nanoparticles modified by LR27. The NPs, PTH/NPs, PTH/LNPs, and PTH/LRNPs were photographed using transmission electron microscopy (TEM). All nanoparticles were spherical with smooth surfaces (Figure 2B). Dynamic light scattering (DLS) revealed average diameters for the NPs, PTH/NPs, PTH/LNPs, and PTH/LRNPs were 151.43 ± 24.17, 152.27 ± 21.66, 158.13 ± 22.73, and 161.57 ± 24.11 nm, respectively (Figure 2C). Meanwhile, the zeta potentials for their surface charges were −1.88 ± 0.20, −0.34 ± 0.19, 0.64 ± 0.25, and 3.09 ± 0.41 mV, respectively (Figure 2D; Table S4, Supporting Information). Although NPs and PTH/NPs exhibited negative surface charges, the absolute value was lower for PTH/NPs than NPs, confirming that PTH1‐34 was successfully loaded into NPs. This trend was consistent with a previous study.^[^ ^28^ ^]^ Moreover, the zeta potentials of PTH/LNPs and PTH/LRNPs were inverted, changing from negative to positive. This was considered to reflect the successful binding of LS16 and LR27 on the PTH/NPs surface. Meanwhile, the larger absolute surface charge value of PTH/LRNPs compared with PTH/LNPs was attributed to the positive charge of Arg8 in LR27. Fourier transform infrared (FTIR) analysis further characterized the four groups. The infrared spectrum of NPs primarily exhibited the typical characteristics of PLGA: C─H stretching vibrations at 2998 and 2946 cm^−1^; the ester carbonyl C ═ O stretching vibration of PLGA at 1745 cm^−1^; the typical absorption peak of the ─CH_2_ bending vibration in the PLGA polymer structure near 1383 cm^−1^; and the stretching vibration of the C─O─C ester ether bond in PLGA at 1270 cm^−1^.^[^ ^29^ ^]^ The infrared spectral characteristics of PTH/NPs did not change significantly, primarily due to PTH1‐34 being encapsulated within the NPs. However, the stretching of absorption peaks at 3355 and 1659 cm^−1^ were related to the O─H, N─H, and C ═ O in PTH1‐34, indicating successful PTH1‐34 loading into the NPs. However, the infrared spectral characteristics of PTH/LNPs and PTH/LRNPs changed significantly due to the covalent binding of LS16 and LR27 on the surface of PTH/NPs. Specifically, PTH/LNPs exhibited a mixed absorption peak of O─H and N─H stretching vibrations from LS16 at 3270 cm^−1^; the C ═ O stretching vibration in the peptide amide group was observed at 1616 cm^−1^; and the N─H bending and C─N stretching vibrations related to peptide primary and secondary amine groups were observed at 1523 cm^−1^. Although the infrared spectral characteristics of PTH/LRNPs and PTH/LNPs were similar, PTH/LRNPs exhibited significant stretching of the absorption peak at 3271 cm^−1^ and a notable increase in the relative intensity of C ═ O stretching at 1616 cm^−1^ and its right shoulder peak. These differences were primarily assigned to the Arg8 in LR27. In conclusion, PTH1‐34 was successfully loaded into NPs, LS16, and LR27 were successfully covalently bound to the surface of PTH/NPs (Figure 2E).

The standard curve for PTH1‐34 was measured by ELISA (Figure 2F). The encapsulation efficiency (EE) of PTH1‐34 by PTH/NPs, PTH/LNPs, and PTH/LRNPs were calculated from the standard curve to be 72.52 ± 3.90%, 72.06 ± 3.96%, 72.28 ± 4.20%, respectively (Figure 2G). The PTH1‐34 cumulative release (CR) curves over 72 h revealed that PTH1‐34 was rapidly released during the first 12 h, with 43.13 ± 3.16%, 28.50 ± 2.74%, and 32.27 ± 2.81% of PTH1‐34 released from PTH/NPs, PTH/LNPs, and PTH/LRNPs, respectively. The release of nanoparticles gradually plateaued after 48 h, with PTH1‐34 releases at 72 h of 77.20 ± 2.25%, 62.37 ± 2.58%, and 61.37 ± 2.50%, respectively (Figure 2H).

Flow cytometry results showed that co‐culturing NPs, PTH/NPs, PTH/LNPs, and PTH/LRNPs with HEI‐OC1 cells for three days rarely induced apoptosis (Figure S3A, Supporting Information), demonstrating good biocompatibility. The survival rates of HEI‐OC1 cells after treatment with NPs, PTH/NPs, PTH/LNPs, and PTH/LRNPs were 95.86 ± 0.72%, 96.56 ± 1.26%, 95.92 ± 0.25%, and 95.68 ± 0.47%, respectively (Figure S3B, Supporting Information).

### 2.2 Characterization of Nanodelivery Systems

**Figure 3:**
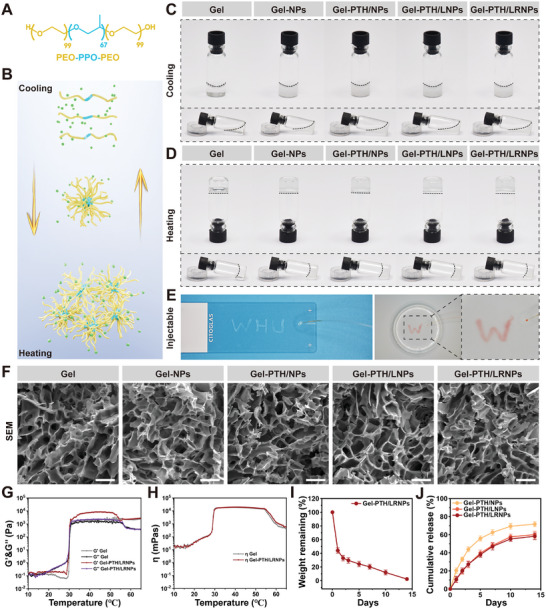
Characterization of different groups of nanodelivery systems. A) Molecular structure to the PEO–PPO–PPEO triblock copolymer. B) Schematic of the sol‐gel transition of F127 loaded with nanoparticles. C, D) Macroscopic views of different groups of nanodelivery systems at low temperatures and body temperature. The black dotted lines represent the surface of nanodelivery systems. E) Demonstration of injectability of Gel‐PTH/LRNPs. F) SEM images of different nanodelivery systems, scale bar: 50 µm. G) Rheological analysis of Gel and Gel‐PTH/LRNPs modulus at different temperatures. H) Viscosity curves of Gel and Gel‐PTH/LRNPs at different temperatures. I) Degradation curve of Gel‐PTH/LRNPs. J) Release kinetics of PTH1‐34 from three nanodelivery system groups. Data are expressed as mean ± SD (*n* = 3).

The F127 thermosensitive hydrogel is a poly (ethylene oxide)‐poly (propylene oxide)‐poly (ethylene oxide) (PEO–PPO–PEO) triblock copolymer. PEO is a hydrophilic block, and PPO is a hydrophobic block (**Figure** **3**A). The reversible transition between the F127 liquid and gel phases was temperature‐dependent. The PEO–PPO–PEO linear molecules were dispersed at low temperatures and formed a physical network at high temperatures (Figure 3B). NPs, PTH/NPs, PTH/LNPs, and PTH/LRNPs were homogeneously dispersed into F127 thermosensitive hydrogels to obtain four groups of nanodelivery systems: Gel‐NPs, Gel‐PTH/NPs, Gel‐PTH/LNPs, and Gel‐PTH/LRNPs. F127 without nanoparticles which named Gel was served as the control.

As demonstrated by the macroscopic images of Gel, Gel‐NPs, Gel‐PTH/NPs, Gel‐PTH/LNPs, and Gel‐PTH/LRNPs in the low‐ and high‐temperature states (Figure 3C,D), F127 without nanoparticles appeared clear, while F127 with nanoparticles appeared turbid. The thermosensitive properties of F127 were not affected by nanoparticles loading. Subsequently, Gel‐PTH/LRNPs were injected onto the slides in warm water (Figure 3E). The Gel‐PTH/LRNPs were injected smoothly without blocking the needle, demonstrating good injectability. As shown in the scanning electron microscope (SEM) images, the nanodelivery systems had porous structures with similar pore sizes and no observable nanoparticles. Hence, the nanodelivery systems exhibited appropriate cross‐linking, and the nanoparticles were well dispersed without significant aggregation (Figure 3F).

The rheological analysis revealed the storage modulus (*G′*) and loss modulus (*G″*) for the different groups of nanodelivery systems (Figure 3G; Figure S4, Supporting Information). A *G′* < *G″* indicated that the gel was liquid, while a *G′* > *G″* indicated that the gel was solid. A larger *G′* corresponded to a greater mechanical strength of the gel. At low temperatures, all groups had a *G′* < *G″*. However, when the temperature gradually increased over 30 °C, *G′* exceeded *G″* and all groups began to form gels. Following gel formation, the *G′* values of Gel‐NPs, Gel‐PTH/NPs, Gel‐PTH/LNPs, and Gel‐PTH/LRNPs were larger than that of Gel. This suggested that F127 loaded with nanoparticles demonstrated a greater mechanical strength. The presence of nanoparticles condensed the physical network of F127. Meanwhile, as the temperature increased beyond 55 °C, *G′* and *G″* decreased for all groups, attributable to water loss from the nanodelivery systems. Additionally, to simulate the modulus change of each group before and after auditory bulb injection, the *G′* and *G″* values were recorded at 4 °C and 37 °C, respectively. (Figure S5, Supporting Information). The viscosities of each group at different temperatures were determined using rheological assays as well (Figure 3H; Figure S6, Supporting Information). F127 loaded with nanoparticles did not exhibit significant differences in viscosity compared with the Gel group. In fact, the viscosity increased most rapidly for each group at ≈30 °C, consistent with the modulus change.

A hydrogel used for middle ear injections must not exhibit excessive mechanical strength as this interferes with auditory ossicle vibrations and causes conductive hearing loss.^[^ ^30^ ^]^ F127 is a hydrogel with low mechanical strength, endowing the formed gel with a soft texture and little effect on the auditory ossicles, which may be advantageous when applied to middle ear injections. The degradation curves of each nanodelivery system group showed the fastest degradation rate on the first day of initiation; the remaining weights of Gel, Gel‐NPs, Gel‐PTH/NPs, Gel‐PTH/LNPs, and Gel‐PTH/LRNPs were 39.33 ± 4.16%, 40.33 ± 3.06%, 42.00 ± 3.61%, 45.33 ± 4.51%, and 44.00 ± 4.00%, respectively, on Day 1. On day 14, all gels were almost completely degraded (Figure 3I; Figure S7, Supporting Information). Additionally, the release of Gel‐PTH/NPs, Gel‐PTH/LNPs, and Gel‐PTH/LRNPs was calculated using a PTH1‐34 standard curve. The CR after 14 days was 71.70 ± 2.78%, 60.07 ± 3.80%, and 58.27 ± 3.69%, respectively. Compared with individual nanoparticles, the release of PTH1‐34 in the nanodelivery systems was smoother and more consistent (Figure 3J).

### 2.3 Biocompatibility of Nanodelivery Systems

**Figure 4:**
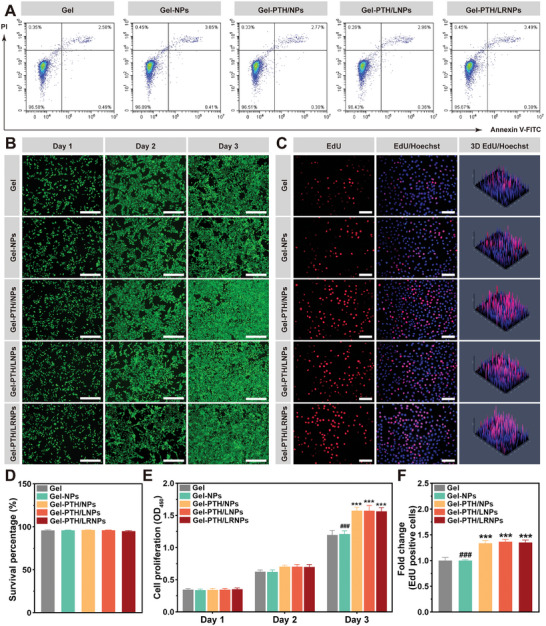
Biocompatibility of nanodelivery systems. A) Flow cytometry images of HEI‐OC1 cells co‐cultured with various nanodelivery system groups for three days. B) Live/dead staining of HEI‐OC1 cells on Days 1, 2, and 3; scale bar: 500 µm. C) EdU staining of HEI‐OC1 cells on Day 3; scale bar: 100 µm. D) Flow cytometric analysis of HEI‐OC1 cell survival rates. E) Relative HEI‐OC1 cell viability measured by CCK‐8 on Days 1, 2, and 3. F) Relative EdU‐positive HEI‐OC1 cells on Day 3 (red). Data are expressed as mean ± SD (*n* = 3). ^***^ *p* < 0.001 compared with the Gel group, ^###^ *p* < 0.001 compared with the Gel‐PTH/LRNPs group.

To assess the biocompatibility of the nanodelivery system, HEI‐OC1 cells were co‐cultured with the various groups for three days and subjected to flow cytometry (**Figure** **4**A). The survival rates of HEI‐OC1 cells after treatment with Gel, Gel‐NPs, Gel‐PTH/NPs, Gel‐PTH/LNPs, and Gel‐PTH/LRNPs were 95.91 ± 0.77%, 95.97 ± 0.27%, 96.30 ± 0.26%, 96.09 ± 0.36%, and 95.04 ± 0.67%, respectively. Compared with the control Gel group, no significant differences occurred among the four groups (Figure 4D). Dead cells (stained red) were rare in all groups, and all live cells (stained green) showed good morphology in the live/dead cell staining images (Figure 4B). HEI‐OC1 cells treated with Gel‐PTH/NPs, Gel‐PTH/LNPs, and Gel‐PTH/LRNPs had greater cell densities on the third day than the other groups, although this trend did not occur on the first two days. Hence, PTH1‐34 promoted HEI‐OC1 cell proliferation. This was further confirmed by the cell counting kit‐8 (CCK‐8) assay over three consecutive days (Figure 4E). The OD_450_ values of Gel, Gel‐NPs, Gel‐PTH/NPs, Gel‐PTH/LNPs, and Gel‐PTH/LRNPs were 0.35 ± 0.02, 0.34 ± 0.02, 0.34 ± 0.02, 0.35 ± 0.02, and 0.35 ± 0.02 on Day 1, 0.63 ± 0.03, 0.62 ± 0.03, 0.70 ± 0.02, 0.70 ± 0.03, and 0.70 ± 0.04, on Day 2, and 1.20 ± 0.07, 1.21 ± 0.05, 1.57 ± 0.05, 1.57 ± 0.08, and 1.56 ± 0.06 on Day 3, respectively. Similar results were obtained in the EdU assay (Figure 4C). Specifically, after standardization with Gel, the relative fold changes of EdU‐positive cells were 1.00 ± 0.01, 1.34 ± 0.05, 1.37 ± 0.04, and 1.35 ± 0.05 in the four groups, respectively (Figure 4F).

Hence, the nanodelivery systems loaded with PTH1‐34 significantly promoted cell proliferation, particularly on Day 3, and exhibited good biocompatibility with HEI‐OC1 cells.

### 2.4 Anti‐Apoptotic Effect of PTH1‐34

**Figure 5:**
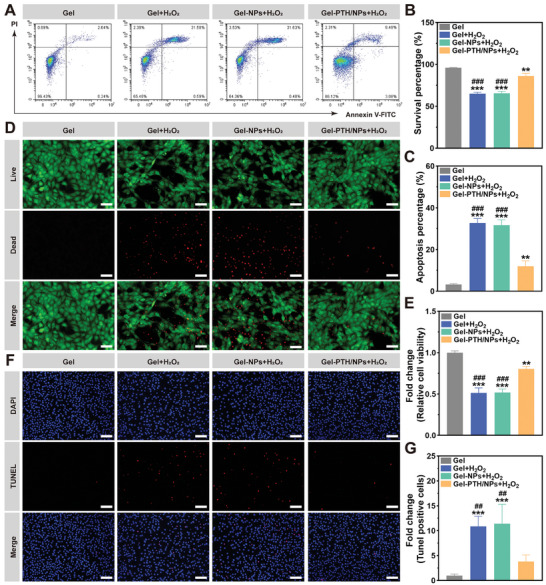
PTH1‐34 inhibits HEI‐OC1 cell apoptosis. A) Flow cytometry images of HEI‐OC1 cells pretreated with different nanodelivery systems for 24 h and co‐cultured with H_2_O_2_ for 8 h. B,C) Flow cytometric analysis of HEI‐OC1 cell survival and apoptosis rates. D) Live/dead staining of HEI‐OC1 cells; scale bar: 50 µm. E) Relative HEI‐OC1 cell viability measured by CCK‐8. F) TUNEL staining of HEI‐OC1 cells (red); scale bar: 100 µm. G) Relative HEI‐OC1 TUNEL positive‐cells. Data are expressed as mean ± SD; (*n* = 3). ^**^ *p* < 0.01 and ^***^ *p* < 0.001 compared with the Gel group, ^###^ *p* < 0.001 compared with the Gel‐PTH/LRNPs group.

NIHL is characterized by hair cell death, with oxidative stress considered the main cause.^[^ ^31^ ^]^ Hydrogen peroxide (H_2_O_2_) is widely used to establish injury models.^[^ ^32^ ^]^ In this study, the relative viability of HEI‐OC1 cells was measured after pretreatment with H_2_O_2_ for 8 h. After standardization against 0 µm, the OD_450_ values of cells exposed to 50, 100, 150, 200, 250, 300, and 350 µm H_2_O_2_ were 0.78 ± 0.05, 0.66 ± 0.05, 0.56 ± 0.04, 0.51 ± 0.02, 0.45 ± 0.02, 0.37 ± 0.02, and 0.29 ± 0.01, respectively. Thus, 200 µm was selected for further experiments as it achieved ≈50% inhibition (Figure S8, Supporting Information). Subsequently, HEI‐OC1 cells were incubated with Gel, Gel + H_2_O_2_, Gel‐NPs + H_2_O_2_, and Gel‐PTH/NPs + H_2_O_2_ to assess the anti‐apoptotic effects of PTH1‐34. Flow cytometry revealed that PTH1‐34 remarkably reversed the injury induced by H_2_O_2_ (**Figure** **5**A), with survival rates increased from 64.98 ± 1.85% in the Gel + H_2_O_2_ group to 86.12 ± 3.15% in the Gel‐PTH/NPs + H_2_O_2_ (Figure 5B). The opposite trend was observed in the apoptosis rate, decreasing from 32.68 ± 2.18% in the Gel+ H_2_O_2_ group to 11.95 ± 2.62% in the Gel‐PTH/NPs + H_2_O_2_ group (Figure 5C).

Live/dead staining confirmed these patterns, with fewer live cells following H_2_O_2_ exposure and compensation achieved by PTH1‐34 (Figure 5D). The images demonstrated that after being treated with H_2_O_2_, many red dead cells appeared and part of them showed morphological abnormalities. In the Gel‐PTH/NPs + H_2_O_2_ group, red dead cells were significantly reduced, and live cell morphology was restored to varying degrees. Moreover, compared to the Gel group, the fold change in cell viability estimated by CCK‐8 was 0.51 ± 0.06 in the Gel + H_2_O_2_ group and 0.80 ± 0.03 in the Gel‐PTH/NPs + H_2_O_2_ group (Figure 5E). Representative TUNEL staining images further confirmed the protective effects of PTH1‐34 (Figure 5F). Compared to the Gel group, the percentage of TUNEL‐positive cells in the Gel + H_2_O_2_, Gel‐NPs + H_2_O_2_, and Gel‐PTH/NPs + H_2_O_2_ groups were 10.87 ± 2.05, 11.42 ± 3.88, and 3.82 ± 1.32, respectively (Figure 5G). These results suggested that PTH1‐34 countered the adverse effects of H_2_O_2_ on HEI‐OC1 cells. The anti‐apoptotic effects of PTH1‐34 in HEI‐OC1 cells under oxidative stress present a potential therapeutic option for NIHL.

### 2.5 Antioxidant Effect of PTH1‐34

**Figure 6:**
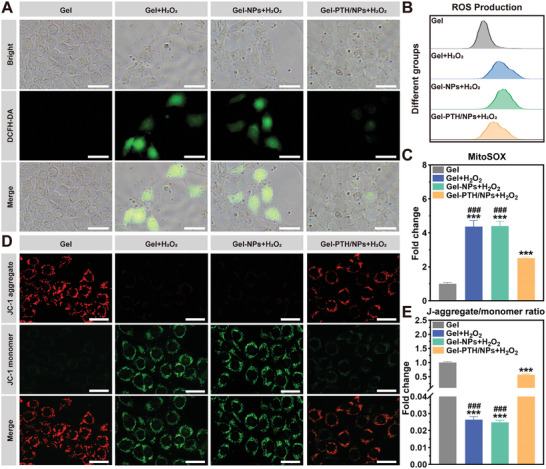
The antioxidant effect of PTH1‐34 on HEI‐OC1 cells. A) DCFH‐DA staining of HEI‐OC1 cells pretreated with different nanodelivery systems for 24 h and co‐cultured with H_2_O_2_ for 8 h; green fluorescence represents ROS; scale bar: 40 µm. B) ROS production and C) mean fluorescence intensity of MitoSOX in cells measured by flow cytometry. D) JC‐1 staining of HEI‐OC1 cells; red: JC‐1 aggregates, green: JC‐1 monomers, scale bar: 40 µm. E) mean fluorescence intensity ratio of JC‐1 aggregate and JC‐1 monomer. Data are expressed as mean ± SD; (*n* = 3). ^***^ *p* < 0.001 compared with the Gel group, ^###^ *p* < 0.001 compared with the Gel‐PTH/LRNPs group.

Overproduction of free ROS is widely recognized as a major mechanism underlying NIHL. Cytoplasmic ROS accumulation may activate apoptotic and necrotic cell death pathways.^[^ ^33^ ^]^ Meanwhile, PTH1‐34 resists oxidative stress and maintains the function of human umbilical vein endothelial cells and bone marrow mesenchymal stem cells.^[^ ^15^ ^]^ Thus, the antioxidant activity of PTH1‐34 was assessed to elucidate the potential mechanism underlying its anti‐apoptotic effects in HEI‐OC1 cells. ROS levels were detected using DCFH‐DA staining (**Figure** **6**A). The level of cellular ROS was significantly weakened after Gel‐PTH/NPs + H_2_O_2_ treatment compared with Gel + H_2_O_2_ or Gel‐NPs + H_2_O_2_. Meanwhile, the bright‐field images confirmed that the cell density and morphology were maintained in the Gel‐PTH/NPs + H_2_O_2_ group. Flow cytometry results were consistent with DCFH‐DA staining (Figure 6B). After standardization against the Gel group, the MitoSOX mean fluorescence intensities of Gel + H_2_O_2_, Gel‐NPs + H_2_O_2_, and Gel‐PTH/NPs + H_2_O_2_ were 4.37 ± 0.36, 4.40 ± 0.28, and 2.51 ± 0.06, respectively (Figure 6C). ROS levels were markedly increased by H_2_O_2_ and ameliorated by PTH1‐34.

Mitochondria serve as the powerhouses of cells, participating in essential metabolic processes.^[^ ^34^ ^]^ ≈90% of all intracellular ROS are produced in mitochondria.^[^ ^35^ ^]^ Overloading of ROS leads to oxidative damage, which increases the permeability of the mitochondrial membrane, disrupting its bioenergetics and leading to mitochondrial dysfunction.^[^ ^36^ ^]^ Thus, JC‐1 staining was performed to evaluate mitochondrial status (Figure 6D). After 8 h of injury, the mitochondria exhibited decreased membrane potential. ROS accumulation in the mitochondria increased and then significantly decreased after PTH1‐34 exposure. The relative JC‐1 aggregate‐to‐monomer ratios were 0.03 ± 0.002 in the Gel + H_2_O_2_ group compared with the control group (1.00 ± 0.03), while that of the Gel‐PTH/NPs + H_2_O_2_ group was 0.57 ± 0.03 (Figure 5E). These data suggested that PTH1‐34 protected HEI‐OC1 cells from oxidative stress by maintaining mitochondrial function and limiting the burst production of ROS.

### 2.6 Protective Effect of PTH1‐34 on Cochlear Explants

**Figure 7:**
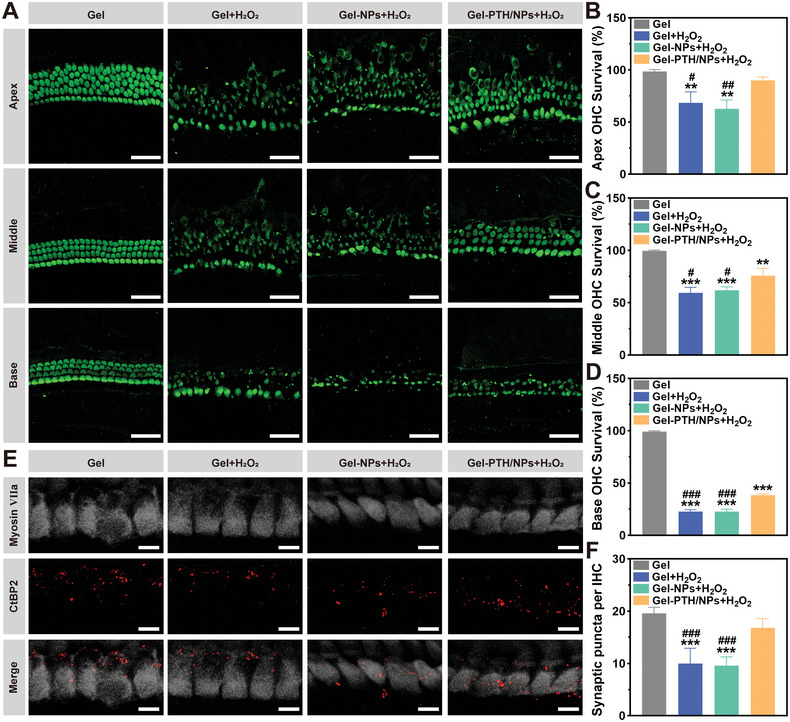
PTH1‐34 treatment attenuates cochlear explant damage induced by H_2_O_2_. A) Immunofluorescence images of cochlear explants pretreated with different nanodelivery systems for 24 h and co‐cultured with H_2_O_2_ for 8 h. Hair cells were stained with Myosin VIIa (green), scale bar: 40 µm. B–D) Quantification of OHCs survival at the apex turn, middle turn, and base turn. E) Immunofluorescence of synaptic puncta on IHCs treated with different groups. IHCs were stained with Myosin VIIa (gray). Synaptic puncta were stained with CtBP2 (red), scale bar: 10 µm. F) The abundance of synaptic puncta per IHC in various groups. Data are expressed as mean ± SD; (*n* = 3). ^**^ *p* < 0.01 and ^***^ *p* < 0.001 compared with the Gel group, ^#^ *p* < 0.05, ^##^ *p* < 0.01, and ^###^ *p* < 0.001 compared with the Gel‐PTH/LRNPs group.

A protective effect of PTH1‐34 on cochlear explants damaged by H_2_O_2_ was observed. Cochlear explants, dissected from P3 suckling mice, were incubated with Gel, Gel + H_2_O_2_, Gel‐NPs + H_2_O_2_, or Gel‐PTH/NPs + H_2_O_2_. Immunofluorescence images of cochlear explants confirmed the survival of outer hair cells (OHCs; **Figure** **7**A). The survival of OHCs after treatment with Gel, Gel + H_2_O_2_, Gel‐NPs + H_2_O_2_, and Gel‐PTH/NPs + H_2_O_2_ were 98.55 ± 1.71%, 68.42 ± 10.60%, 62.80 ± 8.50%, and 90.10 ± 3.05%, respectively, at the apex turn (Figure 7B), 99.49 ± 0.88%, 59.42 ± 5.23%, 61.83 ± 3.34%, and 75.82 ± 7.16%, respectively, at the middle turn (Figure 7C), and 99.15 ± 0.77%, 22.70 ± 1.68%, 22.71 ± 2.21%, and 38.35 ± 1.15%, respectively, at the base turn (Figure 7D). Cochlear explants damaged by H_2_O_2_ exhibited hair cell loss and disorganization. Meanwhile, the survival and arrangement of hair cells treated with Gel‐PTH/NPs were significantly improved. A gradient in the degree of oxidative stress‐induced damage to the cochlear explants was observed. Hair cells were damaged more severely at the base turn than at the apex turn, likely due to the low glutamate levels in hair cells at the base turn and a general lack of antioxidant capacity.^[^ ^37^ ^]^ Additionally, the OHCs were more severely damaged than the inner hair cells (IHCs), partially due to their higher inherent susceptibility.^[^ ^38^ ^]^

Increased oxidative stress leads to the loss of IHCs ribbon synapses.^[^ ^39^ ^]^ Immunofluorescence images of the IHCs ribbon synapses further revealed that Gel‐PTH/NPs exhibited a significant protective role of them (Figure 7E). There were 19.60 ± 1.14, 10.00 ± 2.92, 9.60 ± 1.67, and 16.80 ± 1.79 synaptic puncta per IHC after treatment with Gel, Gel + H_2_O_2_, Gel‐NPs + H_2_O_2_, and Gel‐PTH/NPs + H_2_O_2_, respectively. Ribbon synapses were stained with CtBP2 in this study. Ribbon synapses co‐localized with CtBP2 and GluR2 are typically considered to be functional in adult mouse IHCs. However, GluR2 was difficult to detect using IHCs in P3 suckling mice. GluR2 begins to develop at P10 under the continuous regulation of presynaptic activity.^[^ ^40^ ^]^

### 2.7 Gel‐PTH/LRNPs for NIHL Therapy

**Figure 8:**
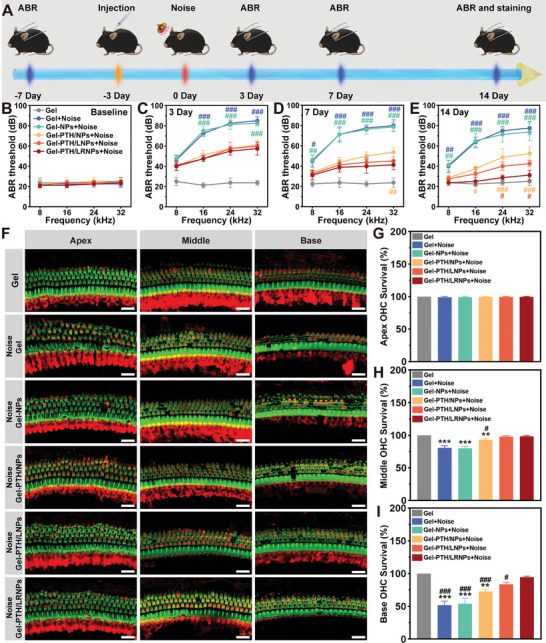
Gel‐PTH/LRNPs for NIHL therapy. A) Animal experiment flowchart. B) Baseline levels of hearing in ABR test mice. C–E) ABR test results in mice on Days 3, 7, and 14 after noise exposure. F) Immunofluorescence staining of hair cells from mice on Day 14 after noise exposure. Hair cells were stained with Myosin VIIa (red) and phalloidin (green), scale bar: 20 µm. G–I) Quantification of OHCs survival at the apex turn, middle turn, and base turn. Data are expressed as mean ± SD (*n* = 4). ^**^ *p* < 0.01 and ^***^ *p* < 0.001 compared with the Gel group, ^#^ *p* < 0.05, ^##^ *p* < 0.01, and ^###^ *p* < 0.001 compared with the Gel‐PTH/LRNPs group.

To further investigate the therapeutic effect of the nanodelivery systems on noise‐induced hearing impairment in vivo, C57BL/6J mice were evaluated based on auditory brainstem response (ABR); those with excellent hearing were selected. Three days before noise exposure, Gel, Gel‐NPs, Gel‐PTH/NPs, Gel‐PTH/LNPs, and Gel‐PTH/LRNPs were injected into the auditory bulbs of mice. Mice were exposed to noise at eight weeks old. After noise exposure and treatment with the different nanodelivery systems, hearing thresholds were tested on Days 3, 7, and 14 by ABR. Mice were sacrificed on Day 14. The cochlea was dissected, and immunofluorescence staining was performed to observe the survival status of OHCs (**Figure** **8**A).

The ABR test showed that the hearing of the experimental mice was normal, with none of the hearing thresholds exceeding 30 dB (Figure 8B). Based on ABR results 3, 7, and 14 days after noise exposure, hearing thresholds did not change significantly after surgery and nanodelivery systems injection into the auditory bulbs, demonstrating the safety of the interventions. The hearing thresholds of mice treated with Gel‐PTH/NPs, Gel‐PTH/LNPs, and Gel‐PTH/LRNPs were reduced to different degrees. The Gel‐PTH/LRNPs group exhibited the best therapeutic effect. According to the ABR results on the third day and after, the mice injected with nanodelivery systems loaded PTH1‐34 had a significantly reduced hearing threshold. This suggested that PTH1‐34 exerted protective effects on the hearing. ABR results of 32 kHz on Day 7 and 8 kHz on Day 14 showed that the mice treated with Gel‐PTH/LNPs and Gel‐PTH/LRNPs had lower hearing thresholds than the Gel‐PTH/NPs group. Hence, the PTH/NPs surface modification by LS16 and LR27 was effective. The powerful function of LR27 was further identified as time passed and the degree of injury increased. ABR results on Day 14 revealed that mice treated with Gel‐PTH/NPs, Gel‐PTH/LNPs, or Gel‐PTH/LRNPs had hearing thresholds 46.25 ± 4.79, 40.00 ± 4.08, and 28.75 ± 2.50 dB at 24 kHz and 52.50 ± 6.45, 42.50 ± 2.89, and 31.25 ± 4.79 dB at 32 kHz, respectively (Figure 8C–E). Thus, Gel‐PTH/LNPs and Gel‐PTH/LRNPs had better therapeutic effects than Gel‐PTH/NPs on hearing loss, with Gel‐PTH/LRNPs eliciting the greatest effect. This was attributed to the fact that Gel‐PTH/LRNPs had hair cell targeting and RWM permeability function, causing a higher PTH1‐34 inner ear transport efficiency.

Immunofluorescence images showed that the loss of OHCs in noise‐injured mice was successfully rescued by Gel‐PTH/LRNPs treatment. The percentages of surviving OHCs injected with Gel, Gel‐NPs, Gel‐PTH/NPs, Gel‐PTH/LNPs and Gel‐PTH/LRNPs were 80.80 ± 3.22%, 80.07 ± 2.99%, 93.48 ± 1.87%, 98.17 ± 1.38% and 98.52 ± 1.18% at the middle turn, and 51.81 ± 6.31%, 54.05 ± 8.37%, 72.83 ± 3.62%, 83.70 ± 2.99%, and 94.60 ± 1.39% at the base turn, respectively. No OHC loss was detected following Gel‐only injection without noise exposure. Similar to the trends of cochlear explants, the cochlear basilar membranes from adult mice were also damaged by oxidative stress in a gradient manner. At the apex turn, minimal hair loss or a disordered hair cell pattern was observed, whereas more severe damage occurred at the base turn, with rows of hair cells lost and hair bundles damaged. Previous studies have revealed that loss of hair cells reduces the amplitude of the basilar membrane, affecting cochlear amplification.^[^ ^41^ ^]^ Meanwhile, damage to the V‐shaped hair bundles at the top of hair cells increases the resistance to lymphatic fluid flow in the inner ear, affecting sound transmission.^[^ ^42^ ^]^ Overall, the multifunctional nanodelivery Gel‐PTH/LRNPs system combined hair cell targeting and RWM permeability to efficiently deliver PTH1‐34 to the inner ear, decrease oxidative stress caused by noise, reduce hair cell damage, and decrease the hearing threshold, achieving satisfactory therapeutic effects.

## 3 Conclusion

In this study, a multifunctional nanodelivery system modified by a fusion peptide named LR27, which acts as a PTH1‐34 carrier, was successfully developed to treat NIHL. The LR27 peptide was assembled by harnessing the advantages of A665, effective in targeting hair cells, and Arg8, with high permeability. The results showed that Gel‐PTH/LRNPs had good biocompatibility with HEl‐OC1 cells and could protect HEl‐OC1 cells from H_2_O_2_‐induced damage by enhancing mitochondrial function to decrease ROS production and ultimately reduce apoptosis in vitro. Gel‐PTH/LRNPs also attenuated oxidative stress‐induced OHCs loss and IHCs ribbon synapse damage in cochlear explants. Moreover, their thermosensitive property rendered Gel‐PTH/LRNPs injectable in the middle ear. Further results demonstrated that PTH/LRNPs penetrated the RWM effectively, and PTH1‐34 was sustained and released into the inner ear to protect hair cells and restore hearing loss induced by noise in vivo. Taken together, these results indicated that Gel‐PTH/LRNPs have great potential for NIHL treatment. Further development of fusion peptides will provide novel strategies for effectively delivering drugs to the inner ear.

## 4 Experimental Section

### Characterization of Peptides

PTH1‐34 was purchased from ProSpec‐Tany TechnoGene (Ness‐Ziona, Israel). Both LS16 and LR27 were purchased from Motif Biotech (Suzhou, China). The purity of LS16 and LR27 was verified via HPLC (LC‐20AT, Shimadzu, Japan). MS (LCMS2020, Shimadzu, Japan) was used to obtain molecular weight information for both compounds.

### Preparation of Nanoparticles

PLGA‐SH (Ruixibio, Xian, China) was fully dissolved in dichloromethane (DCM, Macklin, D807827, China) to obtain a 60 g L^−1^ solution. PTH1‐34 powder and polyvinyl alcohol (PVA, Macklin, P8755084, China) were dissolved in ddH_2_O at 0.5 mg mL^−1^ and 10 g L^−1^, respectively. PTH1‐34 solution (50 µL) was added as an internal aqueous phase to PLGA‐SH solution (1 mL) and mixed by 100 W ultrasonication on ice for 2 min. The obtained emulsion was added dropwise to the PVA solution (20 mL). PVA solution acts as an external aqueous phase, and the mixture was stirred overnight at room temperature to completely evaporate DCM. The next day, the mixture was centrifuged at 4000 rpm for 10 min at 4 °C. The supernatant was collected and centrifuged at 13 300 rpm for 10 min at 4 °C, and the sediment was PTH/NPs. LS16 and LR27 were dissolved in phosphate‐buffered saline (PBS) at pH 7.4. The PTH/NPs were resuspended in LS16 or LR27 solutions and stirred at room temperature to allow the addition reaction to occur overnight. Finally, the sediment was washed thrice with ddH_2_O to remove excess LS16 or LR27. NPs containing ddH_2_O were used as the control. Based on the assembly steps, the four groups of nanoparticles were named NPs, PTH/NPs, PTH/LNPs, and PTH/LRNPs.

### Transmission Electron Microscopy

The morphological characteristics of NPs, PTH/NPs, PTH/LNPs, and PTH/LRNPs were observed using TEM (JEM‐2100, JEOL, Japan). Briefly, the four groups were diluted with ddH_2_O to a suitable concentration and added dropwise to a copper grid. After drying at room temperature, the samples were observed using TEM.

### Average Diameter and Zeta Potential of Nanoparticles

NPs, PTH/NPs, PTH/LNPs, and PTH/LRNPs were dispersed in ddH_2_O at 0.1% wt. and ultrasonicated for 3 min. The average diameter, polymer dispersity index (PDI), and zeta potential were measured with a nanoparticle size and zeta potential analyzer (ZS90, Malvern, Britain).

### FTIR Analysis

NPs, PTH/NPs, PTH/LNPs, and PTH/LRNPs were collected and dried in an oven at 37 °C. They were measured using an FTIR spectrometer (Thermo Scientific, USA) with potassium bromide as the background at room temperature, and the infrared spectral signals of the samples were scanned 32 times per minute in the 4000 to 400 cm^−1^ frequency range with a resolution of 0.5 cm^−1^. The data were processed using OMNIC 8.0 software and the plotting was performed with Origin 2017 software.

### Preparation of Nanodelivery Systems

F127 (Engineering for Life, EFL‐F127‐001) was fully dissolved in ddH_2_O to 20%wt. The NPs, PTH/NPs, PTH/LNPs, and PTH/LRNPs were homogeneously dispersed in F127 at 1% wt. concentration. F127 alone was used as the control. Based on the assembly steps, the five nanodelivery system groups were named Gel, Gel‐NPs, Gel‐PTH/NPs, Gel‐PTH/LNPs, and Gel‐PTH/LRNPs. A digital camera (80D, Canon, Japan) was used to record macroscopic images of each group at different temperatures.

### Scanning Electron Microscopy

The surface morphology of Gel, Gel‐NPs, Gel‐PTH/NPs, Gel‐PTH/LNPs, and Gel‐PTH/LRNPs was observed using SEM. The five samples were rapidly frozen with liquid nitrogen after completely cross‐linking in a 37 °C environment. Subsequently, the samples were dried in a freeze dryer for 24 h to remove the water and held in round aluminum stakes using a double‐sided conductive adhesive for gold spraying for 3 min. SEM (TESCAN, Czech Republic) was applied to examine the surface structures at 20 kV.

### Dynamic Rheological Measurement

The rheological behaviors of Gel, Gel‐NPs, Gel‐PTH/NPs, Gel‐PTH/LNPs, and Gel‐PTH/LRNPs were analyzed using a rotational rheometer (HAAKE MARS 40, Thermo Fisher company, USA). Time and temperature scanning modes, setting the constant strain fixed at 1% and the constant shear frequency fixed at 1 Hz, were used to assess the storage and loss modulus influenced by time and temperature. The shear temperature scanning mode, with a heating rate of 5 °C min^−1^ and fixed shear speed of 10 s^−1^, was used to evaluate the viscosity pattern influenced by temperature.

### In Vitro Degradation

After Gel, Gel‐NPs, Gel‐PTH/NPs, Gel‐PTH/LNPs, and Gel‐PTH/LRNPs initial weights were recorded, they were completely immersed in PBS at 37 °C for 14 days. The weight of each group was recorded on days 1, 2, 3, 5, 7, 10, and 14. The remaining PBS was washed with ddH_2_O and blotted with filter paper before measurement.

### In Vitro Peptide Release

To obtain the release curves of PTH1‐34 in nanoparticles and nanodelivery systems, PTH/NPs, PTH/LNPs, and PTH/LRNPs were immersed in PBS by shaking at 37 °C for 72 h. Gel‐PTH/NPs, Gel‐PTH/LNPs, and Gel‐PTH/LRNPs were immersed in PBS by shaking at 37 °C for 14 days. The supernatant (100 µL) was removed and tested, and the same volume of PBS was replenished. The standard curve (y = 0.0024x + 0.0684) of PTH1‐34 was generated using an ELISA Kit (JINGMEI, JM‐05287H1) at 450 nm. The loaded drug content was defined as the difference value between total drug content and supernatant drug content. The EE and CR were calculated from the standard curve using Equations (1) and (2), respectively:

(1) $$EE=\frac{Total\;drug\;content-Supernatant\;drug\;content}{Total\;drug\;content}\times 100\%$$

(2) $$CR=\frac{Cumulative\;release\;drug\;content}{Loaded\;drug\;content}\times 100\%$$

### Cell Culture

HEI‐OC1 cells were cultured in Dulbecco's modified Eagle's medium (DMEM, Gibco,11995073) with 10% fetal bovine serum (FBS; Sigma–Aldrich, F8318). The incubator was maintained at 33 °C with 5% CO_2_, and the medium was changed every two days. Digestion and passaging were performed when the cell density reached 80–90% using 0.25% trypsin/EDTA (Gibco, 25200056). HEI‐OC1 cells were co‐cultured with different groups of nanoparticles (NPs, PTH/NPs, PTH/LNPs, and PTH/LRNPs) at 1% wt. or nanodelivery systems (Gel, Gel‐NPs, Gel‐PTH/NPs, Gel‐PTH/LNPs, and Gel‐PTH/LRNPs) for 1, 2, and 3 days; their biocompatibility was assessed using CCK‐8 assays, Live/dead staining, EdU staining, and flow cytometry. HEI‐OC1 cells were pre‐treated with Gel, Gel‐NPs, and Gel‐PTH/NPs for 24 h, and co‐cultured with H_2_O_2_ (200 µm) for 8 h. The control group was treated with Gel only during the whole process. Thereafter, apoptosis and ROS production were assessed using CCK‐8 assays, Live/dead staining, DCFH‐DA staining, flow cytometry, TUNEL staining, and JC‐1 staining.

### Cell Counting Kit‐8

CCK‐8 (Vazyme, A311‐02‐AA) was used to assess the viability of HEI‐OC1 cells. Briefly, HEI‐OC1 cells were seeded in 96‐well plates at densities of 3 × 10^3^ and 5 × 10^3^ cells per well to assess cell viability and cytotoxicity, respectively. The CCK‐8 working solution was mixed with FBS‐free DMEM at a ratio of 1:9. Each well was incubated with 100 µL of the mixed solution at 37 °C for 1 h before measuring the absorbance at 450 nm using a multifunctional enzyme marker (HH3500; PerkinElmer, USA).

### Flow Cytometry

The FITC Annexin V kit (BD Biosciences, 556547) was used to assess HEI‐OC1 cell apoptosis. Specifically, HEI‐OC1 cells were digested with EDTA, and the precipitates were collected. The cells were washed twice with PBS (Yeasen, P0315991) and resuspended. FITC Annexin V and propidium iodide (PI) were then added to the cell resuspension solution and incubated for 15 min at room temperature in the dark. The results were analyzed by flow cytometry. ROS accumulation in mitochondria was assessed using MitoSOX Red (Thermo Fisher Scientific, M36008). Briefly, the cells were digested, washed, resuspended, and incubated with MitoSOX Red solution for 15 min at room temperature in the dark. The fluorescence intensity was analyzed by flow cytometry.

### Live/Dead Staining

A live/dead staining kit (Bestbio, BB‐4127) was used to assess cell viability. The staining process was the same as previously reported.^[^ ^43^ ^]^ Briefly, removing the medium and washing twice with PBS, cells were incubated with Calcein‐AM for 15 min at 37 °C in the dark and then incubated with EthD‐1 for 2 min under the same conditions. The residual EthD‐1 was washed using PBS. An inverted fluorescence microscope (IX73, Olympus, Japan) was used to capture images.

### EdU Staining

HEI‐OC1 cell proliferation on day 3 was measured using an EdU Cell Proliferation Detection Kit (Beyotime, C0078S). The EdU stain labeled nuclei with proliferating viability, and Hoechst 33342 labeled all nuclei. An inverted fluorescence microscope was used to obtain images, and EdU‐positive HEI‐OC1 cells were analyzed using ImageJ software.

### TUNEL Staining

HEI‐OC1 cell apoptosis was assessed using a TUNEL staining kit (Beyotime, C1089). Briefly, HEI‐OC1 cells were washed with PBS and fixed with paraformaldehyde (Biosharp, BL539A) for 30 min. After washing with PBS, the cells were permeabilized with 0.3% Triton X‐100 (BioFroxx,1139ML100) for 5 min. Next, the HEI‐OC1 cells were washed twice with PBS. TUNEL working solution was added to the cell culture dish and incubated for 60 min at 37 °C in the dark. Excess TUNEL working solution was washed away with PBS. Nuclei were stained using DAPI (Solarbio, S2110). Images were captured using a laser confocal microscope (Leica STELLARIS 5 SR, Germany). TUNEL‐positive HEI‐OC1 cells were quantified using ImageJ software.

### DCHF‐DA Staining

ROS levels were measured in HEI‐OC1 cells using a ROS Assay Kit (Beyotime, S0033S). Briefly, HEI‐OC1 cells with different treatments were incubated with DCHF‐DA for 20 min and washed with FBS‐free DMEM. Images were captured using an inverted fluorescence microscope.

### JC‐1 Assay

The mitochondrial membrane potential of HEI‐OC1 cells was tested using a mitochondrial membrane potential assay kit with JC‐1 (Beyotime, C2006). Briefly, HEI‐OC1 cells were treated as previously described. The JC‐1 working solution was added to HEI‐OC1 cells and incubated for 20 min at 37 °C. Thereafter, the medium was discarded, and the cells were washed twice with JC‐1 staining buffer (1×). Images were captured using a laser confocal microscope, and the ratio of the average fluorescence intensity of JC‐1 aggregates to that of monomers was measured using ImageJ software.

### Cochlear Explant Culture

C57BL/6J mice were sacrificed at P3. The cochleae were dissected using sterilized scissors and dissecting forceps (Dumont, Switzerland) and placed in cold Hank's balanced salt solution (HBSS, Yeasen, 600147ES76). The volute was carefully opened under a microscope (SOPTOP, China). The modiolus and vasculature were removed. The cochlear explants were transferred to RatCol (Advanced BioMatrix, 5153). Subsequently, they were pre‐treated with Gel, Gel‐NPs, and Gel‐PTH/NPs for 24 h, and co‐cultured with H_2_O_2_ (200 µm) for 8 h. The control group was treated with Gel only during the whole process.

### ABR Test

C57BL/6J mice were placed under deep anesthesia in an RZ6 System TDT workstation (Alachua, FL, USA). The mice were tested at 8, 16, 24, and 32 kHz. Each frequency was measured from 90 dB and decreased by 5 dB until no organized responses were detected. The hearing threshold was determined by the amplitude of wave II.

### Mouse Model of NIHL

Eight‐week‐old male C57BL/6J mice were purchased from SPF Biotech (Beijing, China). All procedures were approved by the Ethics Committee of Zhongnan Hospital of Wuhan University (approval no. ZN2024052). Mice with normal hearing were selected based on the ABR and divided randomly into six groups: Gel (control), Gel, Gel‐NPs, Gel‐PTH/NPs, Gel‐PTH/LNPs, and Gel‐PTH/LRNPs. The surgical procedure for middle ear injection was as follows: hair behind the ears was removed and sterilized. The epidermis was opened longitudinally, and subcutaneous muscles and tissues were bluntly separated to expose the auditory bulb. The auditory bulb was opened using the tip of a needle with a small hole, and cold Gel, Gel‐NPs, Gel‐PTH/NPs, Gel‐PTH/LNPs, or Gel‐PTH/LRNPs were injected. The wound was sutured layer by layer. Three days later, the mice were placed in customized square wire cages (9 cm long) and exposed to 120 dB broadband noise for 2 h, except for the control group. The sound was produced using System RZ6 (TDT). ABR tests were performed on Days 3, 7, and 14 after noise exposure.

### Immunofluorescence Staining

C57BL/6J mice were euthanized 14 days after noise exposure. The cochlea and explant were both fixed with 4% paraformaldehyde. The cochlea was placed into an EDTA decalcified solution (Biosharp, BL616B), which was replaced daily for three days. Subsequently, the sample was permeabilized with 2% Triton X‐100 for 15 min and blocked with 10% donkey serum (Sigma, D9663) for 1 h. Samples were incubated with the primary antibodies of rabbit anti‐Myosin VIIa (Proteus Bioscience, 25–5790; 1:200) at 4° C for 24 h and mouse anti‐CtBP2 (BD Biosciences, 612044, 1:200) at 37 °C for 24 h. Samples were then incubated with appropriate secondary antibodies: goat anti‐mouse IgG1 (Alexa Fluor 594, Thermo Fisher Scientific, A21125,1:1000) at 37 °C for 2 h in the dark, goat anti‐rabbit IgG (Alexa Fluor 350, Thermo Fisher Scientific, A‐11046, 1:200), goat anti‐rabbit IgG (Alexa Fluor 488, Thermo Fisher Scientific, A32731, 1:500), and goat anti‐rabbit IgG (Alexa Fluor 594, Thermo Fisher Scientific, A78956, 1:500) at 4 °C for 24 h in the dark. The cytoskeleton was labeled by phalloidin (Alexa Fluor 488, Thermo Fisher Scientific, A12379, 1:500) at 4 °C for 24 h in the dark. Finally, DAKO (S3023) was added to the sample dropwise and sealed with nail polish. Images were captured using a confocal laser microscope.

### Statistical Analysis

All data were recorded as the mean ± standard deviation (SD), and at least three independent experiments were performed per assay. Statistical analyses were performed using Microsoft Excel 2021 and GraphPad Prism version 9. Student's *t*‐test or one‐way analysis of variance (ANOVA) followed by Tukey's post‐hoc test was used to determine the level of significance. *p* < 0.05 was considered significant.

## Conflict of Interest

The authors declare no conflict of interest.

## Author Contributions

J.L. and Z.H. have contributed equally to this work. Z.H., J.L., and X.C. carried out the conceptualization and experimental design. J.L. and Z.H. performed the majority of the experiments. F.K., Z.L., W.W., and S.W. assisted with experiments related to safety assessment in mice and drug injection surgery. Data analysis and presentation were conducted by Z.H. and J.L. with support from F.W., B.X., and M.H. for data interpretation and presentation. The manuscript was prepared through discussions on data analysis, interpretation, and presentation by Z.H., J.L., S.Z., and X.C., with contributions from all authors.

## Supporting information



Supporting Information

## Data Availability

The data that support the findings of this study are available in the supplementary material of this article.
