# Supplementary material for: A Multifunctional Nanodelivery System Modified by Fusion Peptides Acts as Teriparatide Carrier for Noise‐Induced Hearing Loss Therapy
Source: Adv Sci (Weinh). 2025 Feb 7;12(29):2408798. doi: 10.1002/advs.202408798 (PMC12362802; doi:10.1002/advs.202408798)
Supplement: Supplementary file 1 — Supporting Information [file ADVS-12-2408798-s001.docx]

A Multifunctional Nanodelivery System Modified by Fusion Peptides acts as Teriparatide Carrier for Noise-Induced Hearing Loss Therapy

Jiawen Li^#1^, Zhuowen Hao^#2^, Fangzi Ke^1^, Zhihui Liu^1^, Weilong Wang^1^, Sihui Wen^1^, Fan Wu^3^, Bowen Xu^1^, Miao He^1^, Shengyu Zou^1^, Xiong Chen^*1^, Jingfeng Li^*2^, Zuhong He^*1^

1 Department of Otorhinolaryngology-Head and Neck Surgery, Zhongnan Hospital of Wuhan University, Wuhan, China.

2 Department of Spine Surgery and Musculoskeletal Tumor, Zhongnan Hospital of Wuhan University, Wuhan, China.

3 Department of Pathology and Laboratory Medicine, the Medical University of South Carolina, Charleston, USA.

*Correspondence author: Zuhong He: hezuhong@whu.edu.cn

Jingfeng Li: jingfengli@whu.edu.cn

Xiong Chen: zn_chenxiong@whu.edu.cn

#These authors have contributed equally to this work


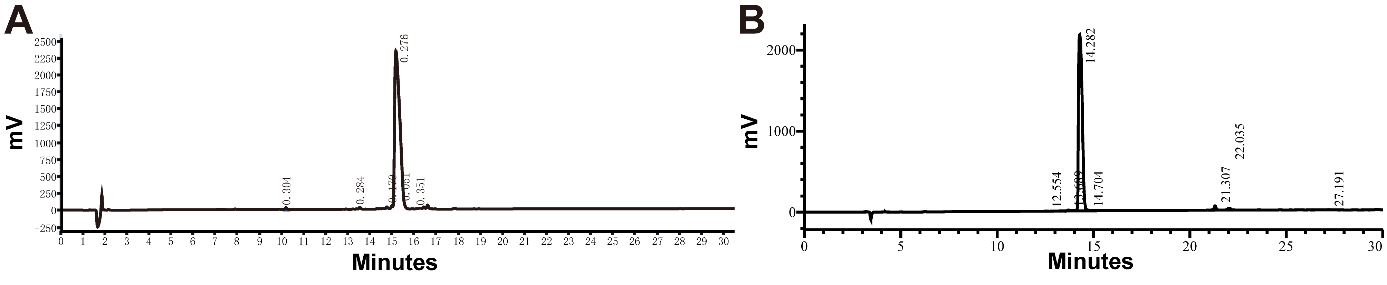


**Figure S1.** The high-performance liquid chromatography (HPLC) peak of (A) LS16 and (B) LR27.

**
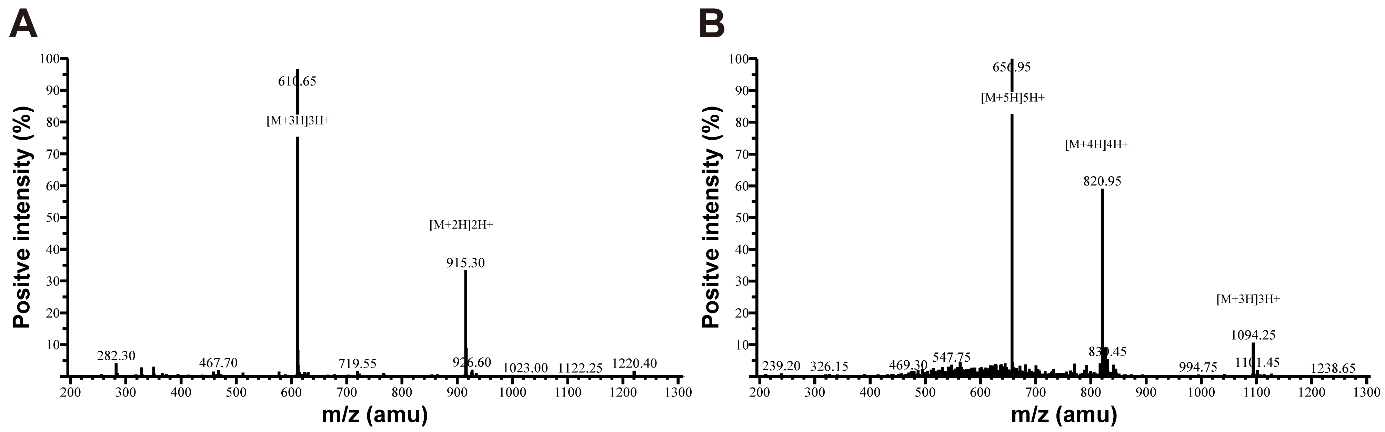
**

**Figure S2.** The mass spectrum (MS) of (A) LS16 and (B) LR27.

**
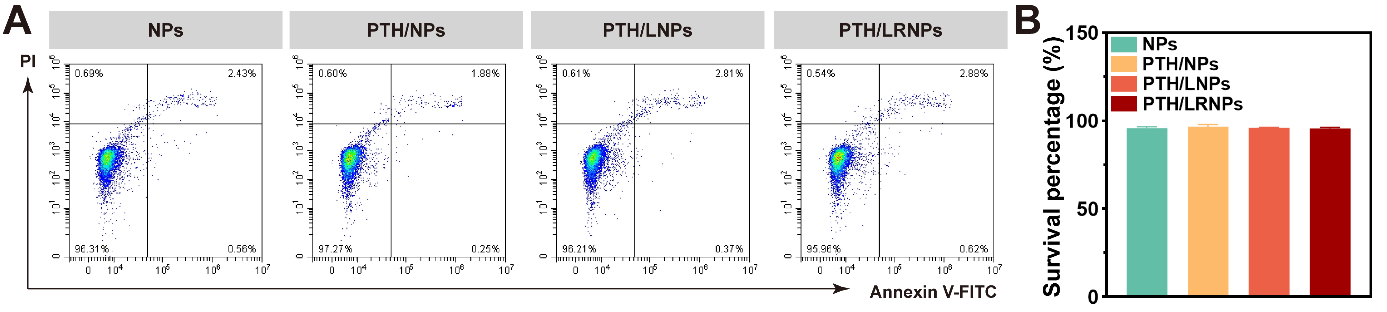
**

**Figure S3.** Biocompatibility of four nanoparticle groups. (A) Flow cytometry images of HEI-OC1 cells co-cultured with various groups of nanoparticles for three days. (B) Survival percentage of HEI-OC1 cells on day 3.


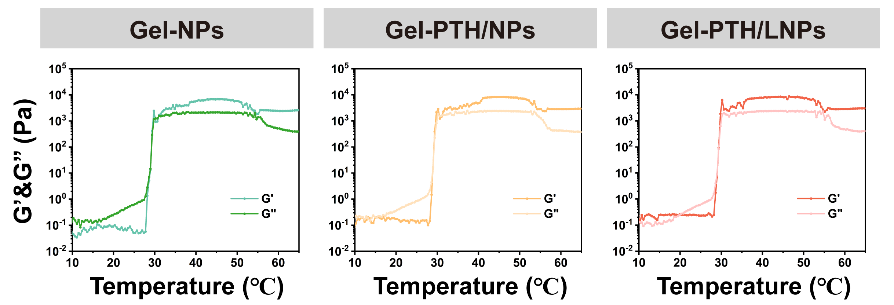


**Figure S4.** Rheological analysis of modulus of Gel-NPs, Gel-PTH/NPs, and Gel-PTH/LNPs at different temperatures.

**
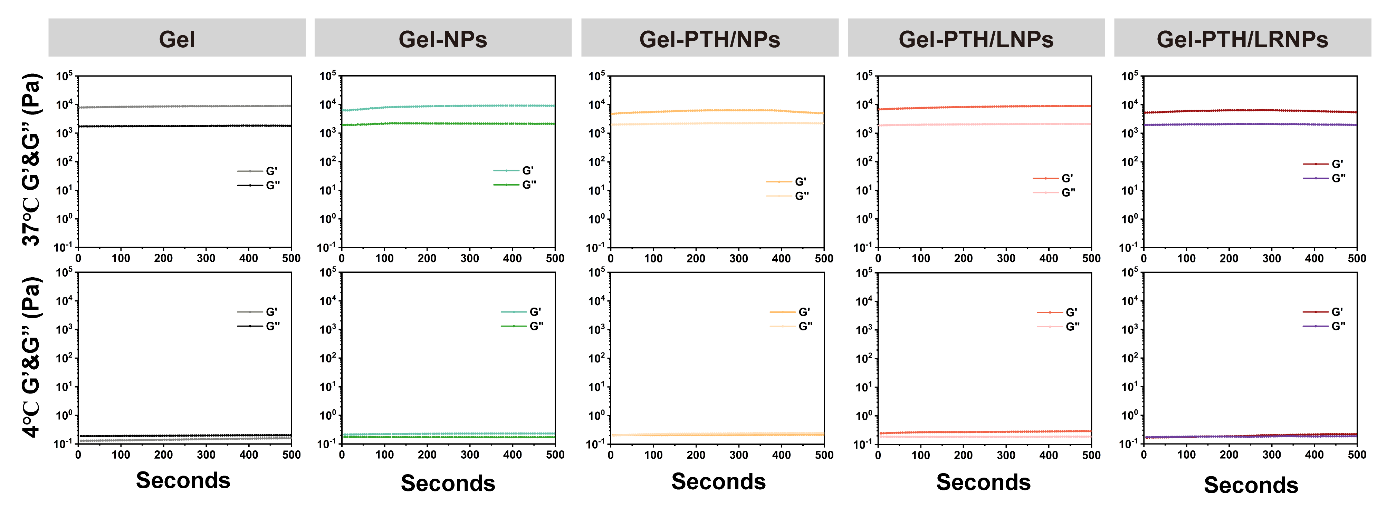
**

**Figure S5.** Rheological analysis of modulus of different groups of nanodelivery systems at 37℃ and 4℃.

**
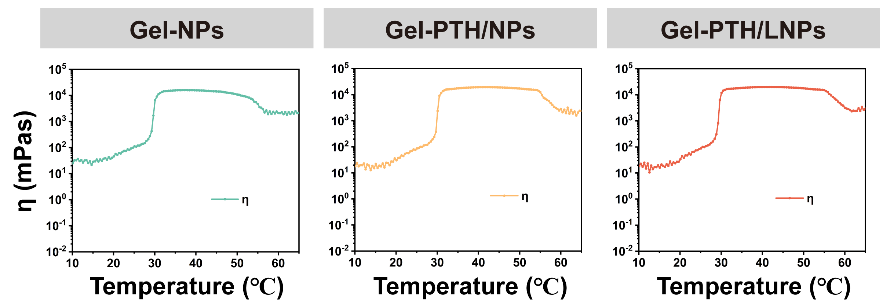
**

**Figure S6.** Viscosity curves of Gel-NPs, Gel-PTH/NPs, and Gel-PTH/LNPs at different temperatures.

**
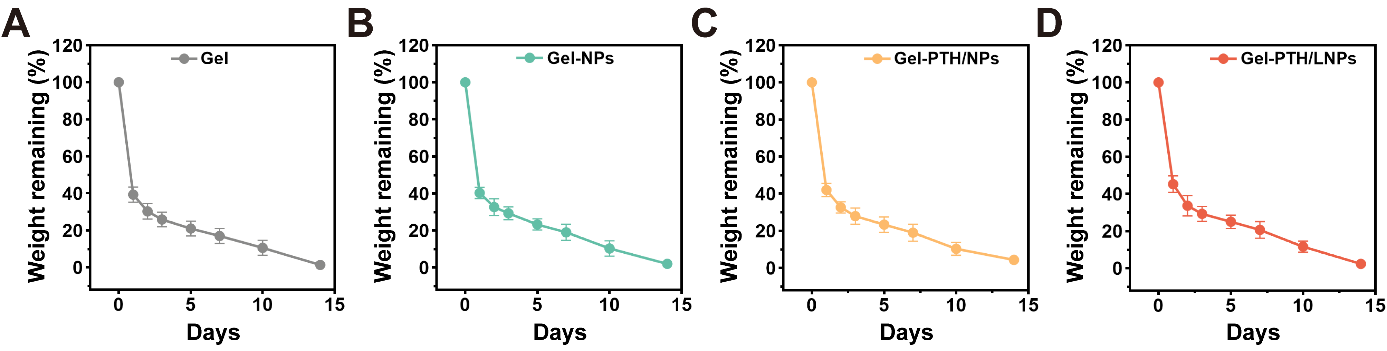
**

**Figure S7.** Degradation curves of Gel, Gel-NPs, Gel-PTH/NPs, and Gel-PTH/LNPs for 14 days.

**
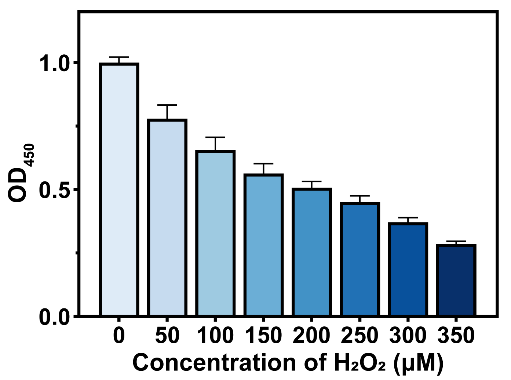
**

**Figure S8.** Relative cell viability of HEI-OC1 cells damaged by H_2_O_2_ at different concentrations for 8 h.

**Table S1.** Names and sequences of peptides

| **Number** | **Name** | **Amino acid sequence** |
| --- | --- | --- |
| 1 | A665 | LSTHTTESRSMV |
| 2 | PrTP1 | LSTHTTESRSMVGGSCGGS[K(N_3_)] |
| 3 | LS19 | LSTHTTESRSMVGGSCGGS |
| 4 | LS16 | LSTHTTESRSMVGGS[K(Maleimide)] |
| 5 | Arg8 | RRRRRRRR |
| 6 | LR27 | LSTHTTESRSMVGGSRRRRRRRRGGS[K(Maleimide)] |

**Table S2.** HPLC peak results of LS16

| **Peak** | **Ret. Time** | **Area** | **Height** | **Area (%)** |
| --- | --- | --- | --- | --- |
| 1 | 10.216 | 129.41 | 25.21 | 0.33 |
| 2 | 13.552 | 161.09 | 27.31 | 0.41 |
| 3 | 15.011 | 653.59 | 50.21 | 1.65 |
| 4 | 15.183 | 37627.52 | 2332.48 | 95.16 |
| 5 | 15.568 | 245.39 | 79.43 | 0.62 |
| 6 | 16.281 | 143.46 | 13.28 | 0.36 |
| 7 | 16.444 | 168.62 | 29.40 | 0.43 |
| 8 | 16.617 | 337.75 | 56.09 | 0.85 |
| 9 | 17.855 | 72.96 | 10.73 | 0.18 |

**Table S3.** HPLC peak results of LR27

| **Peak** | **Ret. Time** | **Area** | **Height** | **Area (%)** |
| --- | --- | --- | --- | --- |
| 1 | 12.554 | 13533 | 1896 | 0.045 |
| 2 | 13.689 | 254983 | 11644 | 0.849 |
| 3 | 14.282 | 28950604 | 2173346 | 96.424 |
| 4 | 14.704 | 150491 | 6402 | 0.501 |
| 5 | 21.307 | 368954 | 51263 | 1.229 |
| 6 | 22.035 | 254130 | 22905 | 0.846 |
| 7 | 27.191 | 31557 | 3858 | 0.105 |

**Table S4.** Characterization of different nanoparticle groups

| **Group** | **Average diameter (nm)** | **PDI** | **Zeta potential (mV)** |
| --- | --- | --- | --- |
| NPs | 151.43 ± 24.17 | 0.22 ± 0.02 | -1.88 ± 0.20 |
| PTH/NPs | 152.27 ± 21.66 | 0.21 ± 0.03 | -0.34 ± 0.19 |
| PTH/LNPs | 158.13 ± 22.73 | 0.24 ± 0.03 | 0.64 ± 0.25 |
| PTH/LRNPs | 161.57 ± 24.11 | 0.21 ± 0.02 | 3.09 ± 0.41 |
